# Tuberculous Meningitis: Immunocompetence, Secondary Immunodeficiency, or Adult Onset Primary Immunodeficiency?

**DOI:** 10.4269/ajtmh.19-0535a

**Published:** 2019-11

**Authors:** Andres Felipe Zea-Vera

**Affiliations:** Faculty of Health; Universidad del Valle; Cali, Colombia; Clinical Immunology Clinic; Hospital Universitario del Valle; Cali, Colombia; E-mail: andres.zea@correounivalle.edu.co

Dear Sir,

I have read with great interest the article “Early Mortality among Immunocompetent Patients of Tuberculous Meningitis: A Prospective Study” published by Jaipuriar et al.,^1^ and I would like to provide some related comments. Frequently, clinicians present case series of adults with extrapulmonary tuberculous infections, using HIV ELISA seronegativity as an equivalent of immunocompetence.

To consider an adult as immunocompetent, clinicians should first rule out secondary immunodeficiency (SID). Among the most relevant causes of SID are infectious diseases (HIV, hepatitis C virus, hepatitis B virus, herpesvirus, and human T lymphotropic virus), metabolic diseases, genetic and systemic diseases (diabetes and kidney and liver diseases), malignancies (hematological and solid organ cancer), autoimmunity (systemic lupus erythematosus or amyloidosis), drugs, radiation, and others.^2,3^ From my point of view, a deeper evaluation is necessary before considering as immunocompetent the patients presented in this article.

Primary immunodeficiency (PID) represents challenges for investigation in tuberculosis-endemic regions.^4^ Chronic granulomatous disease, antibody deficiencies, interleukin-12 (IL-12) receptor deficiency, or interferon-gamma (IFN-γ) and IL-23/IL-17 pathway defects are causes of PID in adults^5^ and could contribute to the severity of tuberculous meningitis. Patients with PID can develop CD4 lymphopenia or dysfunction, which has been associated with severe tuberculosis.^6^ Evaluation of cellular immune response is mandatory, not only CD4/CD8 T-cell quantification (apparently not evaluated in this article) but also functional T-cell responses (e.g., lymphoproliferation and cytokine production). Severe mycobacterial infections in adults can also accompany elaboration of autoantibodies that inhibit cytokines, including anti-IFN-γ in previously healthy adults^7^ or antibodies against IL-17 and IL-22.^8^ This group of autoantibodies is now recognized to cause acquired immune disorders resembling primary genetic immunodeficiency diseases.^9^ Finally, clinical syndromes such as idiopathic CD4 lymphopenia^10^ or secondary lymphopenia are relevant in adults with TB infections and have been associated with severe disseminated infections. In this article, neither cellular nor humoral immune status was evaluated. The term immunocompetent must be used more carefully and in this case should be replaced with HIV-seronegative. The intention of this letter is to generate awareness about the possibility of PID in adult patients with central nervous system TB complications.
